# Effects of Dietary Astaxanthin Supplementation on Energy Budget and Bioaccumulation in *Procambarus clarkii* (Girard, 1852) Crayfish under Microcystin-LR Stress

**DOI:** 10.3390/toxins10070277

**Published:** 2018-07-04

**Authors:** Zhenhua An, Yingying Zhang, Longshen Sun

**Affiliations:** College of Animal Science and Technology, Yangzhou University, Yangzhou 225009, China; zhangyingying@yzu.edu.cn (Y.Z.); lssun@yzu.edu.cn (L.S.)

**Keywords:** microcystin-LR, *Procambarus clarkii*, energy budget, astaxanthin

## Abstract

This research aimed to study the effects of astaxanthin on energy budget and bioaccumulation of microcystin-leucine-arginine (microcystin-LR) in the crayfish *Procambarus clarkii* (Girard, 1852). The crayfish (21.13 ± 4.6 g) were cultured under microcystin-LR stress (0.025 mg/L) and were fed with fodders containing astaxanthin (0, 3, 6, 9, and 12 mg/g) for 8 weeks in glass tanks (350 mm × 450 mm × 150 mm). Accumulations of microcystin-LR were measured in different organs of *P. clarkii*. The results suggested that astaxanthin can significantly improve the survival rate and specific growth rate (SGR) of *P. clarkii* (*p <* 0.05). The dietary astaxanthin supplement seems to block the bioaccumulation of microcystin-LR in the hepatopancreas and ovaries of *P. clarkii* to some extent (*p <* 0.05). Astaxanthin content of 9–12 mg/g in fodder can be a practical and economic choice.

## 1. Introduction

In China, cyanobacteria blooms have often been observed in some large lakes around Jiangsu province. These lakes and nearby ponds are also characterized by the presence of cultured red swamp *Procambarus clarkii* (Girard, 1852). As the most invasive freshwater crayfish in the world, *P. clarkii* is supposed to be tolerant to extreme conditions and is easily cultured, even in water where some cyanobacteria blooms occur [1]. Microcystins, as a group of cyclic polypeptides, are the most common cyanotoxins produced by several genera of cyanobacteria. Several studies have shown that exposure to microcystins can either directly kill organisms or decrease their resistance to bacterial or viral infections [2] and to some extent postpone the creatures’ growth speed. Perhaps that is why *P. clarkii* in nutrient-enriched waters with cyanobacteria blooms always grow slowly and weakly. However, there are also studies suggesting that the *P. clarkii* individuals can feed on microalgae and accumulate toxins in their tissues, without showing any apparent changes in their behavior or vitality [3]. Considering the enormous crayfish consumption in China, this makes the presence of microcystins a serious problem for *P. clarkii* culture and safe consumption in this area.

Astaxanthin, as a useful antioxidant [4] is supposed to be a key composition that can make a great contribution to growth performance, maturation, and carapace color of crayfish [5]. It is also reported that astaxanthin can relieve the negative circumstance stress on juvenile kuruma shrimp *Marsupenaeus japonicus* [6] and astaxanthin contents increased significantly within the interval between the juvenile stages I and II in the embryonic development of crayfish *Astacus leptodactylus* [7]. In this study, the effects of astaxanthin on energy budget and microcystin-leucine-arginine (microcystin-LR) bioaccumulation of the crayfish *P. clarkii* were measured. Our aims were to develop protocols to accelerate the microcystin-LR depuration of crayfish *P. clarkii* and assess the effects of astaxanthin. The results may provide a reference for the astaxanthin promotion in *P. clarkii* cultures.

## 2. Results

### 2.1. Growth

During the experiment, some crayfish stopped wiggling antennae and crouched. The experiment showed that after nearly two months of poisoning, the reactivity and movement of some tested crayfish became slower and they subsequently died. The survival ratios of the experiment are shown in Table 1.

**Figure 1:**
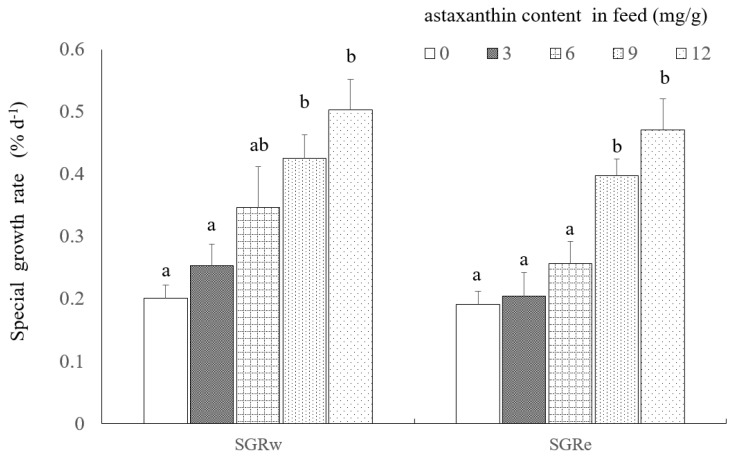
The effects of astaxanthin content (0, 3, 6, 9, 12 mg/g) in feed on the specific growth rate (SGR_w_ and SGR_e_) (expressed as mean ± S.E., *n* = 3) of the crayfish *Procambarus clarkii* (Girard, 1852) under microcystin-LR stress (25 ug/L). Histograms sharing a common letter on top are not significantly different (*p* > 0.05).

The maximum and minimum specific growth rate (SGR) (SGR_w_ and SGR_e_, respectively) occurred in treatment E (12 mg/L astaxanthin concentration ration). Stepwise regression analysis showed that SGR_w_ and SGR_e_ increased with increasing astaxanthin concentration (Figure 1). The relationship among SGR_w_ and SGR_e_ and astaxanthin concentration (Ac %) can be described by the regression equations:

SGR_w_ = 0.191 + 25.867 Ac (*r*^2^ = 0.899, *n* = 5) (1)

SGR_e_ = 0.153 + 25.113 Ac (*r*^2^ = 0.863, *n* = 5) (2)

**Figure 2:**
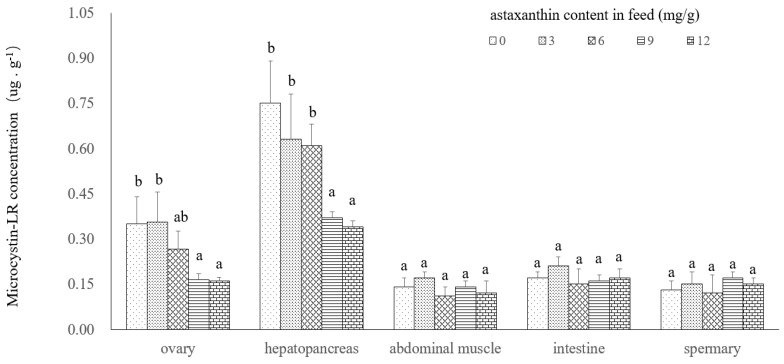
Biological enrichment of microcystin-LR (expressed as mean ± S.E., *n* = 3) in the hepatopancreas, muscle, intestine, ovary and spermary of *Procambarus clarkii* (Girard, 1852). Histograms sharing a common letter on top in the same tissue are not significantly different (*p* > 0.05).

The results of one-way ANOVA analysis showed astaxanthin concentrations in the diet had significant effects on SGR_w_ and SGR_e_ values of *P. clarkii* (*p* < 0.05). The SGR_w_ and SGR_e_ values in treatments D and E were significantly higher than the treatments A and B (*p* < 0.05) (Figure 2).

### 2.2. Microcystin Depuration 

In the experiment, microcystin-LR was observed in all organs/tissues analyzed, but mostly in the hepatopancreas and ovary. The microcystin contents were observed to decrease in the following order: hepatopancreas > ovary > intestine > abdominal muscle (Figure 2). The microcystin-LR contents in hepatopancreas and ovary were significantly higher than others and they can be considered as indicators of microcystin-LR stress. The results in abdominal muscle were slightly different to those of the spermary. The results of one-way ANOVA analysis showed that the astaxanthin concentration in diet had a significant effect on bioaccumulation of *P. clarkii* in the ovary and hepatopancreas (*p* < 0.05).

### 2.3. Energy Allocation

The energy budget in the tested crayfish changed with the different astaxanthin concentrations in diet (Table 2). At treatments E and D the ratio of G/C in treatment were significantly higher than in the other groups. The ratio of F/C in treatments suggested no significant differences between each other (*p* > 0.05). The highest ratio of R/C occurred in treatment A and it was significantly higher than in treatments D and E (*p* < 0.05). 

The results suggested that ratios of G/C and R/C were significantly affected by the astaxanthin concentration in the diet (*p* < 0.05). Due to the different numbers of molts, the ratios of E/C in treatment A, B, and C were significantly lower than D and E (*p* < 0.05).

## 3. Discussion

As an organic toxin, microcystin-LR is difficult to degrade and accumulates easily in some aquatic products [8,9], causing public concern about the pollution problems caused by microcystin. Although microcystins are produced by several genera of cyanobacteria, such as *Microcystis*, *Anabaena*, and *Oscillatoria*, the most commonly reported species is *Microcystis aeruginosa*. This species is inclined to live in relatively quiet waters and forms surface water blooms between summer and autumn [10]. In China, around the Changjiang plain, most *P. clarkii* crayfish are cultured in ponds without fast water pump facilities, and that makes the microcystin problem of *P. clarkii* more serious in this area.

There are many studies suggesting that the sensitivity of aquatic animals to microcystins changes depending on the organism [11], the variant, and the mode of exposure [12]. The content of microcystins in different organs can be an indicator of microcystins stress and the organism can also perform depuration of microcystins to some extent [13]. There are several studies on depuration of microcystins in fish and other aquatic organisms, showing a decrease in microcystin content in several organs (liver and muscle) in a time-dependent manner [14,15]. It has been suggested that the most affected organ with regard to the lipid peroxidation caused by microcystin-LR is the liver [16]. In this study, we used the purified microcystin-LR and found the hepatopancreas and ovary to accumulate with toxins than other organs; and this may be the reason why the occurrence of cyanobacteria blooms in the crayfish culture ponds often results in a fall in production and size of captures. The spermary seems to differ from the ovary and its toxin concentration was almost the same as in the muscles. Considering the abundant lipid content of the crayfish ovary during breeding season [17], this finding may be due to the difference of lipid content and the metabolic regulation mechanisms of *P. clarkii*.

Microcystin exposure has been reported to cause oxidative stress to animals [18,19]. In our research the energy allocated for respiration (R) was significantly decreased with the increasing dietary astaxanthin supplementation (*p* < 0.05). This could implicate that astaxanthin could degrade the oxidative stress caused by microcystin-LR to some extent. There was some research suggesting that astaxanthin could also enhance the specific growth rate and relieve the fresh water-osmotic stress in juvenile kuruma shrimp *Marsupenaeus japonicus* [7]. Some research also reported that an astaxanthin-supplemented diet could not only shorten the molting cycle of the juvenile crustacean but also the postlarval stages of some shrimps such as *Penaeus japonicus* [20]. This research showed the similar results that the energy allocated for molt (E) was significantly higher in the treatments where the crayfish were fed with high dietary astaxanthin supplementation (*p* < 0.05). In sum, astaxanthin could apparently reduce the energy used for respiration and increase the growth and molting ratios in total energy consumed in food.

With the rapidly development of the astaxanthin compositing industry and the falling costs of astaxanthin, it is more and more practical to use the astaxanthin in crustacean culture. Based on the growth and energy allocation results, the appropriate supplement of astaxanthin in feed may be around 10 mg/g. In this way, astaxanthin, which is usually considered responsible for crayfish coloration, will also make the crayfish *P. clarkii* a safer food for human consumption.

## 4. Material and Methods

### 4.1. Source of Animals and Acclimation 

The experiments were conducted from 1 September to 27 October 2015, at the Aquaculture Research Laboratory, Yangzhou University. The crayfish were captured in the suburb of Baoying lake, Yangzhou city and cultivated at a temperature 24 ± 2 °C in several fiberglass tanks with tap water aerated for 48 h, and the water pH adjusted to 7.5 ± 0.5. During the acclimation period, the animals were fed twice a day (at 08.00 h and 18.00 h) with commercial feed provided by Fuyuda corporation (41.70% crude protein, 7.67% crude lipid, 7.89% ash, moisture < 2.70%; energy 21.55 kJ/g dry mass). Aeration was provided continuously and one-third of the water volume in all the experiment tanks was exchanged every day. Dissolved oxygen was maintained above 4.0 mg/L. 

### 4.2. Experimental Design and Procedure

The original pure lyophilized microcystin-LR was bought from Express Technology Co., Ltd., (Beijing, China) and was prepared into 10 mg/L of mother solution with double-distilled water. Microcystin analysis was conducted by ELISA test. The ELISA test kits were bought from J & Q Environment Corporation (Beijing, China). This method had a sensitivity of 0.1 ng/mL. The astaxanthin capsules were brought from Fujian Corona Technology Corporation (Fuzhou, Fujian Province, China). Each capsule contained astaxanthin 60 mg (alga extraction), and soybean oil 440 mg. In making the feed, we squeezed the capsules and mixed the contents well with the same batch commodity feed which was also used in acclimation. The feed slowly absorbed the oil through its capillaries and within 1 h there was no discernible solution in water. We also added a little soybean oil in different feeds to compensate for the energy differences caused by astaxanthin adjunction. 

Finally, five astaxanthin feed concentrations (0, 3, 6, 9 and 12 mg/g; treatments A, B, C, D, and E) were made, and each treatment had three parallel groups. Each parallel group had 12 crayfish. Totally 180 crayfish (with average weight 21.13 ± 4.6 g and male: female = 1:1) were used and there were no significant differences between groups. The acclimation was followed by starvation for 24 h. The initial body weight of each experimental animal was measured. The experiments were carried out in glass aquaria (35 cm width × 45 cm length × 15 cm depth), the solution in each experimental group was over 15 L with 0.025 mg/L microcystin-LR concentration and one-third of the water volume in all the experiment tanks was exchanged every day with siphoned feces and uneaten feed to stabilize the microcystin-LR concentration. Aeration was provided continuously, and dissolved oxygen was maintained above 4.0 mg/L.

During the experiment the animals were fed ad libitum twice a day (08:00 h and 18:00 h) with the feed. The experimental temperature was 24 ± 2 °C. The number of dead crayfish was recorded per 24 h and at finally 22 crayfish (12.22%) were dead in the experiment. At the end of the culture, six living crayfish (half male and half female) were picked up in each parallel group. In total, 18 crayfish were prepared for bioaccumulation test in each treatment. The concentrations of microcystin-LR in the hepatopancreas, intestine, gonads (ovary or spermary), and abdominal muscle of the crayfish were measured by the means of ELISA. The analysis of microcystin content was conducted on samples of similar fresh weight (0.5 g for intestine and spermary; 1.0 g for hepatopancreas, abdomen, and ovaries) obtained by pooling the organs/tissues of three crayfish. Each tissue was tested with three samples in each treatment. The ovaries or spermaries were prepared separately from nine females and nine males. Other tissues were randomly sampled from nine of the former crayfish from which gonads had been obtained. Half the crayfish provided both gonads and tissues (hepatopancreas, abdomen muscle, and intestine) for analysis.

### 4.4. Energy Determination and Estimation of Energy Budget

During the 56-day course of the experiment, the weight of each ration was recorded. Uneaten feed, feces, and molt (exuvia) were separated and removed by siphon to avoid decomposition, dried at 65 °C, shattered, weighed, and kept for analysis of energy and nitrogen content. At the end of the experiment, the animals were starved for 24 h, and then weighed and dried at 65 °C for 48 h and shattered for measurement. The energy contents of the crayfish bodies, feed, and feces were measured with a Parr 6300 Oxygen Bomb Calorimeter (Parr, Moline, IL, USA). The energy budget was calculated by the following equation [21]:

C = G + F + U + R+ E (3)

where C is the energy consumed in food; G is the energy deposited as growth; F is the energy lost in feces; E is the energy lost in molt; U is the energy lost in excretion; and R is the energy used for respiration. The value of C and F can be calculated by the weight of the samples of food intake, feces weight, and energy content per gram. G can be calculated by the following equation:

G = (Fw × Fe) − (Iw × Ie) (4)

where Fw and Iw are final body weight and initial body weight of the crayfish, respectively; Fe and Ie are the energy content per germ of final body and initial body of the crayfish, respectively.

F and E were calculated by the following equation:

F = Pw × Pe;(5)

E = Ew × Ee(6)

where Pw and Ew are final collected feces weight and crustaceous membrane weight of the crayfish, respectively; Pe and Ee are the energy content per germ of feces and crustaceous membrane of the crayfish, respectively.

The nitrogen contents of the crayfish bodies, food, and feces were measured with a Vario Elcube elemental analyzer (Elementar, Germany) at the test center of Yangzhou University. The estimation of U was based on the nitrogen budget equation [22]:

U = (CN − GN − FN − EN) × 24,830 (7)

where CN is the nitrogen consumed from food; FN is the nitrogen lost in feces; GN is the nitrogen deposited in the body; EN is the nitrogen deposited in the molt; and 24,830 is the energy content (J·g^−1^) of excreted nitrogen.

The value of R was calculated by the energy budget equation:

R = C − G − F – U − E (8)

### 4.3. Calculation and Data Analysis

Specific growth rate in terms of weight (SGRw) and energy (SGRe) were calculated as:

SGRw (% day^−1^) = 100 × (ln W_2_ − ln W_1_)/D (9)

SGRe (% day^−1^) = 100 × (ln E_2_ − ln E_1_)/D (10)

where W_2_ and W_1_ are the final and initial wet body weight of the crayfish, respectively; E_2_ and E_1_ are the final and initial body energy of the crayfish, respectively; and D is the duration of the experiment. The data were analyzed by SPSS for Windows (Version 19.0) statistical package (SPSS Inc., Chicago, IL, USA). Inter-treatment differences of survival ratios, SGRw, SGRe, concentration of microcystin-LR, and energy allocation were analyzed with one-way ANOVA followed by post-hoc Tukey multiple range tests. Differences were considered significant if *p* < 0.05.

## Figures and Tables

**Table 1:** The survival ratios of *Procambarus clarkii* (Girard, 1852) fed on a diet with different astaxanthin (Ax) contents (0, 3, 6, 9, 12 mg/g) under microcystin-leucine-arginine (microcystin-LR) stress (25 ug/L) (mean ± S.E).^1^

Ax Content mg/g	0	3	6	9	12
Survival ratio %	77.78 ± 4.81 ^a^	83.33 ± 8.33 ^ab^	86.11 ± 12.73 ^a,b^	94.44 ± 4.81 ^b^	97.22 ± 4.81 ^b^

^1^ Values (expressed as mean ± S.E., *n* = 3) with different letters superscript are significantly different from each other (*p* < 0.05).

**Table 2:** Daily energy budgets of *Procambarus clarkii* fed on diets with different astaxanthin contents (0, 3, 6, 9, 12 mg/g) (mean ± S.E) ^1^.

Astaxanthin Content mg/g	C ^2^	G ^3^	F ^4^	E ^5^	U ^6^	R ^7^
0	4304.24 ± 177.48 ^a^	612.47 ± 95.0 ^a^	1027.15 ± 105.80 ^a^	120.56 ± 14.02 ^a^	182.17 ± 14.02 ^a^	2361.89 ± 146.96 ^b^
3	4413.37 ± 151.21 ^a^	678.24 ± 107.11 ^a^	1079.89 ± 77.55 ^a^	141.24 ± 23.59 ^a^	186.47 ± 13.59 ^a^	2327.53 ± 139.19 ^b^
6	4315.56 ± 147.14 ^a^	845.24 ± 125.24 ^a,b^	1024.54 ± 86.78 ^a^	155.73 ± 16.10 ^a^	196.59 ± 16.10 ^a^	2093.46 ± 170.37 ^a,b^
9	4532.36 ± 284.82 ^a^	1048.12 ± 76.37 ^b^	1263.78 ± 97.73 ^a^	221.47 ± 31.68 ^b^	201.21 ± 21.68 ^a^	1797.78 ± 114.54 ^a^
12	4557.24 ± 147.97 ^a^	1175.11 ± 92.45 ^b^	1243.45 ± 107.73 ^a^	233.45 ± 21.68 ^b^	214.53 ± 21.68 ^a^	1690.70 ± 114.54 ^a^

^1^ Values (expressed as mean ± S.E., *n* = 3) with different letters superscript in the same column are significantly different from each other (*p* < 0.05); ^2^ C (J·g^−1^·d^−1^) = energy consumed in food; ^3^ G (J·g^−1^·d^−1^) = energy deposited as growth; ^4^ F (J·g^−1^·d^−1^) = energy lost from feces; ^5^ E (J·g^−1^·d^−1^) = energy lost from molt; ^6^ U (J·g^−1^·d^−1^) = energy lost from excretion; ^7^ R (J·g^−1^·d^−1^) = energy used in respiration.
